# Experiences with the Liverpool care pathway for the dying patient in nursing home residents: a mixed-method study to assess physicians’ and nurse practitioners’ perceptions

**DOI:** 10.1186/s12904-020-00686-y

**Published:** 2020-11-30

**Authors:** Maartje S. Klapwijk, Natashe Lemos Dekker, Monique A. A. Caljouw, Wilco P. Achterberg, Jenny T. van der Steen

**Affiliations:** 1 Department of Public Health and Primary Care, Leiden University Medical Center, P.O. Box 9600, Leiden, 2300 RC the Netherlands; 2 Marente, Leiden, the Netherlands; 3 Institute of Cultural Anthropology and Development Sociology, Leiden University, Leiden, the Netherlands

**Keywords:** End-of-life care, Liverpool care pathway, Quality of care, Palliative care, Dementia, Nursing home

## Abstract

**Background:**

The Liverpool care pathway for the dying patient (LCP) is a multidisciplinary tool developed for the dying phase for use in palliative care settings. The literature reports divergent experiences with its application in a nursing home setting related to its implementation and staff competencies. The aim of this study is to understand how the LCP is being used in the context of the nursing home, including for residents with dementia, and experienced from the perspectives of those responsible for medical treatment in nursing homes.

**Methods:**

A mixed-methods approach was used, consisting of a survey followed by interviews. A link to a 9-item online survey with closed and open-ended questions was emailed to all physicians and nurse practitioners of 33 care organisations with nursing homes in three regions of the Netherlands (North, West and South). In addition, 10 respondents with particularly positive or negative experiences were selected for semi-structured interviews.

**Results:**

The survey was completed by 159 physicians and nurse practitioners. The respondents were very positive on the content and less positive on the use of the LCP, although they reported difficulties identifying the right time to start the LCP, especially in case of dementia. Also using the LCP was more complicated after the implementation of the electronic health record. The LCP was judged to be a marker of quality for the assessment of symptoms in the dying phase and communication with relatives.

**Conclusion:**

An instrument that prompts regular assessment of a dying person was perceived by those responsible for (medical) care to contribute to good care. As such, the LCP was valued, but there was a clear need to start it earlier than in the last days or hours of life, a need for a shorter version, and for integration of the LCP in the electronic health record. Regular assessments with an instrument that focusses on quality of care and good symptom control can improve palliative care for nursing home residents with and without dementia.

## Background

In the last days of life of nursing home residents, the focus often shifts from optimizing quality of life towards optimizing quality of dying. Identifying and managing symptoms such as pain and dyspnoea becomes paramount, in addition to care for relatives, addressing possible spiritual needs, and other needs that people may have in these last days or hours [1, 2].

The ‘Liverpool care pathway for the dying patient’ (LCP) is a multidisciplinary tool that was developed in the United Kingdom (UK) and introduced in hospices in 1997 [3]. It aims to improve care in the last days of life by facilitating decision making and improving communication between the care team and relatives and organizing the care that is needed. Over the last decades, the LCP has been introduced in other countries including the Netherlands, where it was implemented nationally by the Netherlands Comprehensive Cancer Organisation (IKNL) in 2009 [4–6].

The LCP is supposed to start when the patient is expected to die within a few days, and is initiated as this is agreed upon by the multidisciplinary team (typically a physician or nurse practitioner and a member of the nursing staff). The Dutch version of the LCP (in Dutch: Zorgpad Stervensfase, translated: Care pathway for the dying phase) starts with criteria that can help make this decision: the patient is bed bound, is semi-comatose, is only able to take sips of fluids and/or no longer able to take tablets. The LCP consists of three parts. The first part contains items regarding the patient’s physical condition, how to improve comfort, and preferences regarding religious and spiritual needs. In this part, the patient and relative are assessed on awareness of diagnoses and the impending death. Part 2 prescribes regular assessment of symptoms such as pain and dyspnoea carried out every 4 h. Any symptom assessments and other actions by health care professionals (physicians and nursing staff) should be recorded in this part. Part 3 assesses care for the relatives and communication regarding procedures after death. (Examples of care goals in supplement 1).

In the UK, the original instrument for use in hospices was also used to improve care for people dying in hospitals or at home. Inadequate implementation by staff with little understanding of palliative care in these settings led to assumptions that the instrument was used to hasten death and to deprive people of food or fluids. The national outrage this caused resulted in its withdrawal and it was no longer used in the UK after 2012 [7–9].

In the literature divergent experiences have been described, ranging from positive experiences of care professionals and relatives regarding involvement in end-of-life care to criticism based on findings that indicate that the LCP is not adapted for certain groups, such as persons living in nursing homes and people with dementia [10, 11]. Research into validity and reliability of the assessments in the nursing home setting is limited. A 2017 review article by Husebo et al. on the research done in nursing homes on adaptation and validation on the LCP shows that while several studies have addressed the use of the LCP in this setting, no studies were identified that addressed adaption of the LCP to improve fit with the nursing home setting, that no randomized controlled trial, prospective or blinded studies were done in this setting, and no studies were found specifically describing strategies for evaluation of medication, nutrition/hydration or clinical recommendations. Studies that have addressed the use of the LCP in nursing homes outside the UK, have focused on the perceptions of professional and informal caregivers regarding the LCP, suggesting that the LCP is perceived to improve regular assessment of symptoms, as well as communication between and among care professionals and family [12–17].

In 2009, a paper version of the LCP was introduced in the Netherlands and a digital version was issued in 2014 [18]. Three versions with the same content are available; for the home setting, the hospital and the nursing home. There are two differences; first, the nomenclature for the person, which is patient in the hospital version, resident in the nursing home version and client in the home care version. Second, the frequency of symptom assessment with six times a day (every four hours) recommended in the hospital and nursing home versions and four times a day (morning, afternoon, evening, night) in the home care version.

In nursing homes, the educational level of nursing staff (registered nurses, and levels comparable to certified nursing assistants and nurse aids) is mixed but generally lower (only 17% registered nurses in nursing homes) than in hospice and hospital settings with mainly registered nurses [19]. The difference in medical and nursing education, and therefore experience, may impact on the use of the instrument and interpretation of the observations by both nursing and medical staff. Information on the current use and experience with the LCP from the perspectives of those responsible for medical care in nursing homes is therefore vital. Further, in the Netherlands, at the end of life, 61% of nursing home residents have dementia and therefore it is important to better understand the usefulness of the LCP for residents with dementia or cognitive impairment [20]. They are often incapable of verbally expressing their needs, for instance when they are in pain. Therefore, specific pain indicators are available based on research in pain observation in dementia, and a single pain item in the LCP may not acknowledge developments in this field of research [21]. Therefore, a better understanding is needed as to how the LCP is being used and evaluated in practice for nursing home residents including those with dementia, from the perspectives of those responsible for medical treatment in nursing homes.

## Methods

We used a mixed-methods design, combining results from an online survey that solicited for quantitative and qualitative data, with qualitative data gathered in semi-structured interviews. Based on earlier ethnographic research [15] and clinical experience we developed the online survey, with 9 questions regarding knowledge of and experiences with the LCP. This type of research does not fall under the scope of the Medical Research Involving Human Subjects Act (WMO) in the Netherlands. The protocol was reviewed by the Scientific Committee of the department of Public Health and Primary Care of the Leiden University Medical Center. In compliance with the General Data Protection Regulation (GDPR) it was sent via an internet link to three Academic Networks of Elderly Care in the North (UNO-UMCG, 17 care organizations), West (UNC-ZH, 11 care organizations) and South (AWO-ZL, 7 care organizations) of the Netherlands. Academic Networks are networks of nursing home organizations linked to a university medical center with a specific goal to stimulate teaching, research and best-practices in long-term care [22]. Nursing home care in the Netherlands can be defined as 24/7 care for care dependent with on-site nurses and medical staff [23]. Seven care organizations did not respond.

The coordinator of each academic network sent the survey to the coordinator of the nursing home organizations affiliated with the network, who in turn, sent it to all physicians, physician assistants, nurse practitioners, including those in training. Four organizations provided only one or two completed surveys, but they did not report how many people they sent the link to. A total of 499 practitioners received the internet link to the online survey.

In the Netherlands, certified elderly care physicians, physician assistants and nurse practitioners are part of the nursing home staff and deliver most of the medical care to residents. The physicians, physician assistants and nurse practitioners together with a member of the nursing staff agree upon the decision when to start the LCP. The first section of the survey asked about professional specialization, gender, age, number of years of experience working in a nursing home, the organization and the type of unit they are working in, all exclusive to nursing homes. The survey subsequently inquired about experiences working with the LCP, and its availability and motivation for use. The survey was discontinued for respondents who reported they did not know the LCP. Finally, we included questions related to which items respondents would want to keep or change in the LCP, whether they thought nursing staff had enough knowledge about palliative care, and whether this influenced the effect of the LCP. Most items featured a multiple-choice format, some were open-ended. (Survey in supplement 2) The responses were entered online by the participants and managed in Castor EDC, version 2019.2.8.

The data were processed anonymously, but at the end of the survey participants were asked to indicate if they would allow the researchers to contact them for a brief semi-structured interview. Two researchers (MK, elderly care physician and NLD, anthropologist) selected ten interviewees with particularly negative or positive experiences with use or content of the LCP to best understand divergent perspectives. The number of ten was based on literature regarding sample size in qualitative interview studies needed to achieve saturation with otherwise fairly homogenous samples such as those involved in medical care of nursing home residents dying with dementia in the Netherlands [24, 25]. The interviews were recorded with permission; next, they were transcribed as input for thematic analyses. The interview guide covered four questions about the practical use of the LCP such as whether it was used alongside the medical record or if it replaced the medical record as originally intended. Specific questions based on the responses to the open-ended items in the survey were intended to elicit details about the negative or positive experiences they reported in the survey and reasons as to why they felt the LCP was or was not a valuable instrument to improve end-of-life care. Furthermore, the use of other instruments for the dying phase and perceptions of the nursing staff-knowledge of palliative care were explored. Sampling of diverse viewpoints was prioritized above gender distribution. The interviews were semi-structured to give interviewees the opportunity to explain their experiences and the interviewers the opportunity to probe for increased depth if needed. The questions concerned were all LCP-related rather than patient-related or personal. We expected that asking physicians and other health care professionals who frequently manage care for dying people about use of a care path in general would not induce unmanageable levels of stress. All interviewees had completed the survey before and indicated they would volunteer for an additional interview. Health care providers can be expected to be able to reflect on whether considering the topic in the survey would induce any distress and not volunteer for a subsequent interview. We emphasized that the interview can be stopped at all times, and the interviewers would be able to refer to after care if needed.

For thematic analysis, the open-ended survey items along with the interviews were all independently coded by NLD and MK and in part by JTS. All answers to each question in the survey and interviews were coded. An open coding method was used for the interviews. First, the authors independently coded answers by breaking down the answers into relevant fragments and codes. The authors than compared resulting codes and the data was further categorized into themes. The respondents’ characteristics were described based on descriptive analysis with the statistical program SPSS Inc., version 24, IBM, USA.

## Results

The online survey was accessed 159 times; by 103 elderly care physicians, 29 nurse practitioners, 8 general practitioners (2 were also elderly care physician), 18 medical school graduates, 2 physician assistants, and 1 healthcare psychologist. The professionals in training are specified in Table 1. The majority of the respondents were women (70%). The mean number of years of experience was 12 years (standard deviation 10.6 years). Most respondents worked on a psychogeriatric (dementia) unit (91%), many also worked on various other units. Many respondents (50%) were (also) working in a hospice or palliative care unit.

**Table 1:** Baseline characteristics of the study population, 159 respondents

Profession		
Elderly care physician, n (%)	103^*a^	(65)
*Of whom in training, n*	*17*	
*Of whom specialized in rehabilitation, n*	*3*	
*Of whom specialized in dementia care, n*	*2*	
*Of whom specialized in palliative care, n*	*9*	
General practitioner (GP), n (%)	8^*^	(5)
*Of whom in training*	*2*	
Medical School Graduate n (%)	18	(11)
Nurse practitioner n (%)	29	(18)
*Of whom in training*	*2*	
Physician assistant n (%)	2	(1.3)
Healthcare psychologist, n (%)	1	(0.6)
**Gender, n (%)**		
Female	111	(70)
**Age category, n (%)**		
20–30 years	21	(13)
31–40 years	37	(23)
41–50 years	44	(28)
51–60 years	42	(26)
61 or older	15	(9)
**Mean number of years of experience (SD)**	12.0 (10.6)	
Response by region, n (%)^*b^		
UNC-ZH (West), 11 organizations	87	(57)
AWO-ZL (South), 7 organizations	21	(14)
UNO-UMCG (North) 17 organizations	45	(29)
**Units in organization of practice, n (%) (more possible)**		
Psychogeriatric (dementia) unit(s)	144	(91)
Unit(s) for chronically ill	134	(84)
Geriatric rehabilitation unit(s)	117	(74)
Hospice/palliative care unit(s)	79	(50)
Social gerontology/Geriatric psychiatry unit(s)	64	(40)
Unit(s) for people with young-onset dementia	49	(31)
Other unit(s)^*c^	43	(27)
**Units practitioners’ practice, n (%) (more possible)**		
Psychogeriatric (dementia) unit(s)	105	(66)
Unit(s) for chronically ill	71	(45)
Geriatric rehabilitation unit(s)	42	(26)
Hospice/palliative care unit(s)	18	(11)
Social gerontology/Geriatric psychiatry unit(s)	7	(4)
Unit(s) for people with young-onset dementia	9	(6)
Other unit(s)^*c^	22	(14)

*SD* Standard deviation, *UNC-ZH* Universitair Netwerk voor de Care Sector Zuid Holland, *AWO-ZL* Academische werkplaats Ouderenzorg Zuid Limburg, *UNO-UMCG* Universitair Netwerk Ouderenzorg Universitair Medisch Centrum Groningen

^*a^Total number education 161, 1 respondent was both an elderly care physician and a GP, 1 respondent was both an elderly care physician in training and a GP

^*b^Missing = 6 organizations

^*c^e.g. Huntington dis., Parkinson dis., acquired brain injury, short stay

Table 2 shows responses from the 118 respondents (79%, nine missing answers) who indicated knowing the LCP. Availability and readiness to use of the LPC were higher than its actual use in all units. For example, in 39% of the psychogeriatric units the LCP was available, but it was used in only 29%; and it was available in 54%, but only used in 44% of hospice/palliative care units (Table 2).

**Table 2:** Experiences with the Liverpool care pathway (LCP; *n* = 118 respondents who reported to know the instrument)

LCP available and ready to use *(more responses possible, % units in organization indicated in* Table 1*)*		
Psychogeriatric/dementia unit	56	39
Unit for chronically ill	51	38
Geriatric rehabilitation unit	30	26
Hospice/palliative care unit	43	54
Gerontopsychiatric unit	22	34
Young onset dementia unit	20	40
Other	9	21
Not available	31	
**LCP actually used,** *(more responses possible, % units in organization indicated in* Table 1*)*		
Psychogeriatric/dementia unit	42	29
Unit for chronically ill	35	26
Geriatric rehabilitation unit	16	14
Hospice/palliative care unit	35	44
Gerontopsychiatric unit	14	22
Young-onset dementia	13	27
Other	9	21
Not ready to use	26	
Available but not used	22	
LCP format in use^*a^		
Paper version	23	21
Digital version	46	41
Both paper and digital versions	10	9
Neither version	33	30
Experiences with content LCP^*b^		
Mainly positive	85	77
Mainly negative	3	3
No experience	22	20
Experiences with use LCP^*c^		
Mainly positive	67	62
Mainly negative	19	18
No experience	22	20
Missing	10	
In your organization is the knowledge level of nursing staff regarding palliative care sufficient to be able to see positive or negative effects of the use of the LCP^*d^		
Knowledge sufficient, and this supports effect of LCP	31	30
Knowledge sufficient, but does not support effect of LCP	12	11
Knowledge insufficient, but does not affect the effect of LCP	11	11
Knowledge insufficient, and this affects the effect of LCP	24	23
No experience with LCP	27	26

^*a^Missing *n* = 6

^*b^Missing *n* = 8

^*c^Missing *n* = 10

^*d^Missing *n* = 13

The paper version LCP was used by 21% of the respondents, 41% used the digital version, while 9% used both (30% neither). Mainly positive experiences with *the content* of the LCP were reported by 72% of the respondents and only 3% had mainly negative experiences. Regarding *the use* of the LCP, 62% reported mainly positive experiences, and 18% mainly negative while 20% had no experience at all. Of the 118 respondents who indicated knowing the LCP most elaborated on their responses in the open-ended items (77 on experiences with *the content*, 79 with experiences on *the use*, 88 on adaptations and 98 on what to keep). Regarding the last question, related to the level of palliative care knowledge of nursing staff in the organization, a total of 41% of participants answered that this knowledge was sufficient and 34% that it was insufficient. In 23% of the cases the answer was that the knowledge of nursing staff was insufficient and that this impacted the effect of the use of the LCP.

### Interviews

From the 42 respondents who gave permission to be contacted for an interview, we selected ten respondents. We interviewed six elderly care physicians, of whom one was in training, and four nurse practitioners, of whom one was in training. Only one of these was negative on both the LCP content and its use. All others were positive on content; therefore we selected four respondents who held negative viewpoints on use only. (Table 3).

**Table 3:** Characteristics of the interviewees

		Interviewee number									
	n	1	2	3	4	5	6	7	8	9	10
**Profession**											
Elderly care physician (in training)	6	X					X	X	X	X	X
Nurse practitioner or physician assistant (in training)	4		X	X	X	X					
**Gender**											
Female	7	X	X		X	X		X	X		X
Male	3			X			X			X	
**Age category, years (%)**											
20–50 years	5		X	X				X	X		X
51 or older	5	X			X	X	X			X	
**Content of LCP positive + or negative -**		+	+	+	+	+	+	+	+	–	+
**Use of LCP positive + or negative -**		+	–	–	–	+	+	+	+	–	–

Three themes emerged from the analysis of the interviews and the open-ended survey questions [1]: Timing: a need to identify the right moment to start the LCP [2], Changing use of the LCP over time in response to digitalisation, and [3] The use of the LCP as a marker of quality.

### Theme 1: timing: need to identify the right moment to start the LCP

The moment the multidisciplinary team recognizes that the resident is expected to die within the next days is the moment the LCP should be started. Identifying this moment can actually be very difficult, as many respondents and interviewees indicate. The LCP offers some guidance in this. Several interviewees observed that in a nursing home setting it is more difficult to determine if a person is dying because this period can take longer, from weeks to months. The gradual decline complicates decisions as to when to start the LCP.

- *‘It’s a little strange to say, the dying phase starts now. It is often a kind of gradual process. At a certain point your treatment focusses on comfort and wellbeing anyway. Quite often you have agreed on such a palliative policy, and things deteriorate slowly but surely, and the intake also diminishes slowly but surely.’ (interview 1, elderly care physician)*

Another problem mentioned was the relevance of the subjects and questions in part 1 of the LCP. Nursing home residents are frequently admitted for the long term, and stay for several months or years. They often have cognitive impairments or dementia. Many respondents mentioned that part 1 consisted of too many questions, some of which were irrelevant, especially for people with dementia. Others thought the questions were relevant but should be asked earlier, before the dying phase, to help improve care. Furthermore, this information was often already available in the electronic health record and duplicating it was considered a waste of time.

*-'Some people, in my eyes, when you see that someone is going into a phase, he only deteriorates. The difficulty is that we have all these people with dementia who all die here with us sooner or later. But we all have this moment that you say, now we are really in a phase that we are going to approach things symptomatically and we just, when a person gets sick then things go wrong. Some of those steps should already be taken in that phase. And maybe even sooner than that.’ (interview 9, elderly care physician)*

Some respondents felt that being clear about the resident’s medical condition and acknowledging that the person was going to die helped the team and relatives. Others felt that declaring the start of a dying phase was slightly artificial, and it was important to only do this when they were absolutely certain. The participants felt uncomfortable about starting the LCP and then having to withdraw it if the person turned out not to be dying in the next days. Wanting to be absolutely sure that the person would be dying soon, and to avoid confusion among relatives motivated nursing staff to start the LCP relatively late. One respondent even expressed hoping that the expectation of the person dying soon would be met:

*- ‘I sometimes suspect that that, I see it sometimes in different situations around dying, that this fear is there, that once you have said that the end seems to be drawing near, that you hope this expectation is met, so you don’t confuse people.’ (interview 4, nurse practitioner)*

### Theme 2: changing use of the LCP over time in response to digitalisation

Many respondents were bothered by the change in use of the LCP after the introduction of the electronic health record. Before, the LCP (paper version) was used together with the paper version of the electronic health record, or was available in the resident’s room. However, with the introduction of the electronic health record, the practical use of the LCP changed. Some organizations digitalized the LCP and included it in the electronic health record, while others have a separate system. Many respondents claim that the previous advantage of visibility of a paper version either in the health record or in the resident’s room has been lost. The interviewees shared solutions to be able to continue using the LCP as it was, such as scanning the forms completed in ink and including them in the electronic health record.

*-‘I thought it was quite inconvenient that you had to open it separately, and so you don’t have the overview anymore. Quite often before you start up that Care Pathway it’s like, have there been any more discussions, and you are used to one electronic health record, you know all its ins and outs, and I just like to have everything in one, also because when a person eventually dies, the Care Pathway would be closed and, like, scanned, so ultimately it does end up in the record, but in that sense too late. If the family want to follow up on something, then for me it was simply less practical to have to search in two different systems.’ (interview 2, elderly care physician)*

Other problems mentioned as a result of this change in practice was uncertainty as to what to report in which system and the necessity to report in two systems, both the electronic health record and a separate LCP. Several other difficulties mentioned were: a variable availability of the LCP on different wards, but no reasons were given for the system not being available in the whole organization. Uncertainty about the actual use of the LCP and what to complete when, especially if information was not available yet; often respondents indicated not knowing whether they filled in the LCP correctly; it took too much time to complete all items. It would also take time to re-familiarize themselves with the items, as the LCP was used infrequently.

Overall, the respondents were very positive about the content of the LCP and less positive on its use; many would prefer a less complicated instrument that is integrated in the actual digital system. Integrating the LCP in the usual way of working on the ward would also enable users to use it in a more proactive way and keep an overview of all the information needed, such as medical history and actual use of medication, including during the night or weekend.

*-‘I personally wasn’t very impressed with it, but that was primarily because it was not integrated into the electronic health record and so I would regularly run into that the team used it as they should, but subsequently did not report anything, so I would be unable to anticipate anything at all during my shift.’ (interview 9, elderly care physician)*

### Theme 3: the use of the LCP as a marker of quality

Another important theme mentioned in the survey and interviews was quality of care. The need to care for a dying person and to make sure that the symptom burden is as low as possible increases the relevance to promptly recognise and treat symptoms. Many physicians and nurse practitioners were positive about the concept of regular symptom assessment. They wanted the dying person to be assessed regularly and felt that the LCP was an appropriate tool. The interviewees referred to the comprehensiveness and structure of the pathway and that it made nursing staff more aware of the process of dying and the need to provide more comfort in this phase. Many described the regular assessment as the main motive to continue working with the LCP. One of the interviews also highlighted that the LCP increased awareness for less common symptoms. No other end-of-life instruments were used when asked in the interviews, apart from a pain instrument that was mentioned once.

*‘I think that it is a reminder for the physician and nursing staff that those symptoms in particular should also receive attention. There are some symptoms that are better known or more obvious and then there are some ‘poor relations’. And if these do not get attention, or they are never reported.’ (interview 2, elderly care physician)*

The importance of good communication between nursing staff and physician was also acknowledged as contributing to quality. Some respondents had concerns about nurses not always recognizing all the symptoms and residents being treated late for some symptoms as a consequence. Another respondent thought the knowledge of the nursing staff on care in the dying phase is adequate but that some fellow physicians responded insufficiently to signals from the nursing staff regarding symptom management.

*‘Yes I think the knowledge is there, absolutely. Certainly among the nursing staff, and in my shifts I encounter stories about arrogant doctors who do not listen to nurses and who think the suffering observed by the nursing staff is not that bad, and I have regularly come across situations that I think, well, they could have started better symptom treatment sooner and more adequately.’ (interview 1, elderly care physician)*

Clear communication about the actual expected death and informing relatives and colleagues was named as a positive aspect of the LCP. Also adding to the quality of care was the possibility to literally show the relatives that their loved one was on the LCP, by placing the paper version of the LCP in the resident’s room, visualizing for the relatives that they were completing forms. The LCP was felt to reassure the relatives that the team was working very diligent.

*‘Yes, also that the due care is there, that symptoms are closely monitored and also reported, yes that provides the family with a sense that staff work diligently here.’ (interview 10, nurse practitioner)*

One respondent mentioned the name of the LCP and its introduction in the Netherlands by the IKNL (Netherlands Comprehensive Cancer Organisation), indicating that it is only for cancer patients, and suggested an instrument be developed for nursing home residents. However, the same person appreciated the completeness of the LCP and stated that almost all questions are also relevant in the nursing home setting. Especially the awareness of the dying phase and the heightened alertness to possible discomfort in this phase were often mentioned in the answers. Some felt that the LCP’s contribution to quality of care was largest in teams where knowledge on palliative care was insufficient, and that the LCP might add less in a setting with more experience with dying, such as a hospice. One respondent added that the *increase* in quality diminishes as use of the LCP becomes more frequent.

Another concern raised was the risk of the LCP being used as a checklist and the specific knowledge necessary to recognize pain or shortness of breath being lost. These symptoms can go unrecognized, while the boxes can still be ticked.

*-‘Yes and I also feel it is important that there is something, that everyone has a kind of checklist, like have we done everything now? What I said, some doctors give little information, some nurses give little information and then the family are in a constant state of stress and tension, while this could easily be done differently. On the other hand there are also situations where everything is so easy, so gradual, that the whole list, at some point it is like a checklist and then it feels a bit bureaucratic to me.’ (interview 9, elderly care physician)*

Use of the LCP by nursing staff reassured the physicians that a sudden change in symptom burden would not be missed by the health care team, and nurses would learn about the importance of monitoring symptoms. Related to nursing staff being poorly educated in identifying and managing symptoms and physicians not being fully able to remedy this problem, the physicians would favour the opportunity to improve quality of care for the dying with the LCP, the only instrument they knew.

## Discussion

This study shows an overall positive perspective on the part of many of the respondents on the use and content of the LCP. Some points of critique were found, mainly regarding the use in the electronic health record format. Another important outcome of this study is the need to start an end-of-life pathway in the nursing home setting at an earlier stage and to connect a pathway to the knowledge and care goals that are already available in the electronic health record. Many respondents indicate the necessity of an instrument that can be used in the dying phase, but point out that it is difficult to find the right moment to start the LCP. They are reluctant to start the LCP too early and then have to withdraw it, which leads to late starts of the LCP. The requirement of agreement within the multidisciplinary team can also delay the start of the LCP. This means its use and possible benefits are available for an even shorter period of time, which implies there is room to improve the quality of end-of-life care.

The four criteria in the LCP that can help the multidisciplinary team decide if a person is in the dying phase are extra difficult to apply to people with dementia. They have often already been bedridden, drink very small amounts and are no longer capable of taking tablets for longer periods. This is certainly true for people in a more advanced stage of dementia [26]. So in those cases three of the four criteria are not helpful to determine if a person is in the dying phase.

Several respondents indicated that the usual care in a nursing home is already focused on comfort and well-being, and that this focus of care does not change after the start of the LCP. Although several studies show no clear evidence regarding effectiveness of the use and outcome of the LCP [27, 28], many respondents agreed on the helpful structure to improve communication. This is in line with the findings from earlier studies in nursing home settings [11–13, 15]. Interestingly, while these studies also point to possible improvements in symptom management, not one respondent in our study mentioned results related to earlier or better symptom control.

Although the use of the LCP lead to a positive view among 77% of the respondents, one might question the quality of the assessments as guided by the LCP instrument. Research on assessment of symptoms has shown it can be difficult to interpret symptoms such as pain, especially in people that may have difficulty verbally expressing themselves, such as people with dementia [29]. Some indicated the risk of the LCP becoming a list of boxes to be ticked off, a risk already pointed out in connection with the use of the LCP [8].

Use of the LCP as a marker of quality, to the organization and to the relatives was found to be a motivation to use the pathway. Many respondents also indicated that use of the LCP improved communication within the care team and with the relatives.

Another important issue was the impact of repeated use of the instrument. Would more frequent use result in more benefits for the resident in a linear fashion with no ceiling effect, or would quality increase the most when it is used infrequently by inexperienced care staff? Other research showed that it was more difficult to work with the LCP when it was not used frequently [15]. It is important to teach care staff how to work with an instrument and use it in the intended manner [8, 30]. This is even more important when, as in the case of dementia, recognizing symptoms is already extremely challenging.

The interviews showed that the respondents did not use other end-of-life pathways alongside or as a replacement of the LCP. This lack of pathways for end-of-life care in the nursing home setting is worrying and may indicate room for improvement through implementing instruments tailored to the nursing home setting.

The LCP was developed to transfer principles of hospice care to other settings such as hospitals and nursing homes to improve care for people dying [31]. One of the important lessons from the critique and withdrawal of the LCP in the UK is the clear need for adequate education and implementation. In Dutch nursing homes, the (elderly care) physician or nurse practitioner start the LCP together with a member of the nursing staff when they both believe a resident is dying. Compared to hospital and hospice settings, nursing home residents are cared for during a longer period of time which may facilitate recognizing changes in health status and communication to prepare for dying. At that point, wishes regarding end of life have often already been discussed with the resident or their family caregiver and this may lower the risk of inappropriate use of the LCP. The withdrawal of the LCP in the UK did not lead to a national debate in the Netherlands. However, the results of this study together with the clear lessons and recommendations from the Neuberger review [32] regarding communication and involvement in a care plan indicate that there is room to improve the LCP for the nursing home population.

### Strengths and limitations

This mixed-methods study presents the results of the use and experiences of the LCP reported electronically by a large number of respondents who were reached via a link sent to various organizations. Due to the GDPR we could not collect email addresses to send individual invitations to participate in the survey. This is also why we could not determine the exact number of persons who received the link, but almost 90% of the contacted organizations informed us about how the link had been distributed. We approached three Academic Networks of Elderly Care in the Netherlands to include different parts of the country. It is possible that the associated organizations are used to work more with pathways and tools than other organizations and therefore the results may not be representative for the Netherlands. The focus in this study was on those responsible for medical treatment in nursing homes, therefore we did not collect data from the perspective of the nursing staff and the relatives.

Nevertheless, we believe that the high number of respondents reflects a relevant perspective on the actual use in Dutch nursing homes in different regions in the country. This is the first study to give an insight into actual use and application of the LCP. We were surprised to find that 20% of the respondents were not familiar with the LCP. The positive but also negative perspectives reported in the interviews added valuable information to complement the results of the survey.

### Benefits and limitations of the LCP in practice

Many respondents recognized benefits of the LCP in that it facilitated communication within the team and with relatives. It also reassured physicians that the patient was being monitored. As such, and mainly through regular symptom assessments and the importance of also involving an educational component, the LCP was experienced as a marker of quality. This motivated continuing the use of the instrument. Disadvantages referred to administrative burden, practical limitations in recording on paper or digitally in more systems and use merely as a tick-off exercise that did not really help to improve quality. Research in six European countries showed that knowledge of nurses and care assistants concerning basic palliative care issues was variable but suboptimal in all participating countries [33]. Future projects could use this information and also focus on (repeated) training and educational programmes in nursing homes, with the aim to improve communication between nursing staff and physician.

### Implications for practice

Overall, this study shows that practitioners who are responsible for the medical treatment in the nursing homes feel a need for a care pathway. This pathway should be integrated in the electronic health record to better support anticipation, recognition and treatment of symptoms. There is also a need to start such a pathway at an earlier stage, so as to improve palliative care not only in the last days or hours of life, but in the last weeks to months, and to make it more applicable to the nursing home population, which includes people with dementia. Regular evaluation of care goals is necessary, and instruments such as the IPOS-Dem or use of heuristics for nursing staff [34, 35] can be used to improve quality of palliative care for people with dementia. Frequent symptom assessment can be performed several times a day when death is expected within weeks or days.

## Conclusion

This mixed-methods study with 159 survey respondents and ten interviews provides an understanding of how the LCP is being used and experienced in practice for nursing home residents, including those with dementia. Those responsible for (medical) care perceived an instrument that prompts regular assessment of a dying person as contributing to good care. As such, the LCP was valued, but there was a clear need to start it earlier than in the last days or hours of life-perhaps related to many residents having dementia. There was also a need for a shorter version and for integration of the LCP in the electronic health record. Such regular assessments with an instrument that focusses on quality of care and good symptom control can improve palliative care for nursing home residents with and without dementia.

## Supplementary Information

**Additional file 1.** Examples of care goals per section of the Liverpool care pathway.

**Additional file 2.** Survey regarding the use and experiences of the Liverpool care pathway.

## Data Availability

The datasets generated and analysed during the current study are not publicly available due not having explicit permission from survey respondents and interviewees to provide access; however, the survey data from the respondents who choose to not provide contact details are anonymous and are available from the corresponding author on reasonable request.

## Abbreviations

LCP: Liverpool care pathway for the dying patient
UK: United Kingdom
IKNL: Integraal Kanker Centrum Nederland or Netherlands Comprehensive Cancer Organisation
GDPR: General Data Protection Regulation
