# Supplementary material for: Experiences with the Liverpool care pathway for the dying patient in nursing home residents: a mixed-method study to assess physicians’ and nurse practitioners’ perceptions
Source: BMC Palliat Care. 2020 Nov 30;19:183. doi: 10.1186/s12904-020-00686-y (PMC7706263; doi:10.1186/s12904-020-00686-y)
Supplement: Supplementary file 1 — Additional file 1. Examples of care goals per section of the Liverpool care pathway. [file 12904_2020_686_MOESM1_ESM.docx]

**Supplement 1 Examples of care goals per section of the Liverpool care pathway**

**Source:** Geijteman EC, Dekkers AG, van Zuylen L. [10 years after implementation of the Liverpool Care Pathway for the dying patient; important improvements in end-of-life care]. Nederlands tijdschrift voor geneeskunde. 2013;157(37):A6174.

**Part 1: Assessment at start of dying phase**

-the resident and his or her family know that the resident is dying

-the treatment team have the correct contact information for the family

-the resident and his family have been given the opportunity to discuss what is important to them at that moment, e.g. regarding keeping watch, religious/spiritual customs or rituals

-currently prescribed medication has been assessed and unnecessary medication discontinued

-if necessary, medication has been prescribed for symptoms that may occur in the final days to hours

-a syringe driver is available to enable continuous or intermittent administration of medication

-an infusion needle has been placed subcutaneously for intermittent subcutaneous administration of medication

-current interventions have been assessed and unnecessary interventions discontinued, e.g. routine blood tests and assessment of vital functions

-a do-not-resuscitate policy has been agreed; any internal defibrillator has been deactivated

-involved health care professionals have been informed that the resident is dying, e.g. the GP, specialist or specialists, and paramedics

**Part 2: Assessment of resident-related problems**

-the resident is pain free (move resident only for comfort)

-the resident’s breathing is not hindered by secretions (in case of rattling, repeated explanations to the family if necessary)

-the resident has no micturition problems (consider inserting a urinary catheter in case of retention, incontinence or weakness)

-when administering medication, the safety and comfort of the resident are ensured (check the syringe driver and the insertion site of the subcutaneous infusion needle at least once every 4 h)

-attention is paid to the psychological well-being of the resident (explain interventions and care, attention for communication and religious/spiritual support)

-attention is paid to the well-being of those close to the resident (among other things, explain the possible symptoms of the resident and what they mean, and ascertain the needs of the persons staying with the resident)

**Part 3: Care after death**

-the institution's procedures after death have been followed, e.g. the resident has been cared for with dignity and respect and the spiritual, religious and cultural needs of the resident and family have been met

-involved healthcare providers have been informed of the death of the resident, such as the GP, the specialist or specialists, and paramedics
