# Supplementary material for: Experiences with the Liverpool care pathway for the dying patient in nursing home residents: a mixed-method study to assess physicians’ and nurse practitioners’ perceptions
Source: BMC Palliat Care. 2020 Nov 30;19:183. doi: 10.1186/s12904-020-00686-y (PMC7706263; doi:10.1186/s12904-020-00686-y)
Supplement: Supplementary file 2 — Additional file 2. Survey regarding the use and experiences of the Liverpool care pathway. [file 12904_2020_686_MOESM2_ESM.docx]

**Supplement 2 Survey regarding the use and experiences of the Liverpool care pathway**

1.1 You are: (more than one answer possible)

- Elderly Care physician

- General Practitioner

- Nurse practitioner

- Medical resident in training to become elderly care physician

- Medical resident in training to become general physician

- Nurse practitioner in training

- Elderly Care physician with Special Interests - Rehabilitation

- Elderly Care physician with Special Interests - Psychogeriatrics

- Elderly Care physician with Special Interests - Palliative care

- Medical School Graduate

- Other, namely: ……….

1.2 You are:

- Female

- Male

1.3 Your age:

- 20-30 years

- 31-40 years

- 41-50 years

- 51-60 years

- 61 years or older

1.4 Number of years of experience in the nursing home: please fill in the number of years and/or months (only whole numbers, no decimal points please)

- .. years and/or …. months

2.1 You work in an organization that is part of one of the Academic Networks of Elderly Care

- UNC-ZH, Leiden (University Network for the Care Sector)

- UNO-UMCG, Groningen (University Elderly Care Network- University Medical Centre Groningen)

- AWO-ZL, Maastricht (The Living Lab in Ageing & Long-Term Care Zuid-Limburg)

2.2 You work within the organization (UNC-ZH): (please fill in)

- ………………………………….

2.2 You work within the organization (AWO-ZL): (please fill in)

- ………………………………….

2.2 You work within the organization (UNO-UMCG): (please fill in)

- ………………………………….

2.3 You work at the following location(s): (please fill in)

- ………………………………….

3.1 Can you indicate what types of units your care organization consists of: (more than one answer possible)

- Psychogeriatric (dementia) unit(s)

- Unit(s) for chronically ill

- Social gerontology/Geriatric psychiatry unit(s)

- Geriatric rehabilitation unit(s)

- Hospice/Palliative care unit(s)

- Unit(s) for people with young-onset dementia

- Other unit(s), namely………..

3.2 Can you indicate at what types of units you are working as regular physician/nurse practitioner? (more than one answer possible)

- Psychogeriatric (dementia) unit(s)

- Unit(s) for chronically ill

- Social gerontology/Geriatric psychiatry unit(s)

- Geriatric rehabilitation unit(s)

- Hospice/Palliative care unit(s)

- Unit(s) for people with young-onset dementia

- Other unit(s), namely………..

4. Are you familiar with the Liverpool c**are pathway**?

- No: I am not familiar with the Liverpool **care pathway**. (If you do not know the Liverpool **care pathway** you can select this answer and then close the questionnaire by closing the link.) This is followed by the message: Thank you for your cooperation!

- Yes, I am acquainted with the Liverpool **care pathway** (please continue with the next question).

5.1 Is the Liverpool **care pathway** AVAILABLE and ready for use in the care organization where you work? (more than one answer possible)

- Yes; in the psychogeriatric unit(s)

- Yes; in the unit(s) for chronically ill

- Yes; in the social gerontology/geriatric psychiatry unit(s)

- Yes; in the geriatric rehabilitation unit(s)

- Yes; in the hospice/palliative care unit

- Yes; in the unit(s) for people with young-onset dementia

- Yes; in other unit(s), namely: ………….. (please enter below)

- No; it is not available

5.2 1 Is the Liverpool **care pathway** actually being USED in the care organization where you work? (more than one answer possible)

- Yes; in the psychogeriatric unit(s)

- Yes; in the unit(s) for chronically ill

- Yes; in the social gerontology/geriatric psychiatry unit(s)

- Yes; in the geriatric rehabilitation unit(s)

- Yes; in the hospice/palliative care unit

- Yes; in the unit(s) for people with young-onset dementia

- Yes; in other unit(s), namely: ………….. (please enter below)

- No; it is not ready for use; please indicate below why the care pathway cannot be used.

- No; it is available, but not used (everywhere). If you select this answer, please indicate the reason why it is not being used: ……

6. If the Liverpool **care pathway** is being used, is this a paper version or a digital version of the **care pathway**?

- Paper version

- Digital version

- Both

- No use of care pathway

7.1 Are the experiences you have with the CONTENT of the Liverpool **care pathway** mainly positive or mainly negative?

- Mainly positive experiences

- Mainly negative experiences

- No experience using **care pathway**

7.2 Please describe your most important experiences with the CONTENT of the Liverpool **care pathway**

-……………………………………………….

7.3 Are your experiences with the USE of the Liverpool **care pathway** mainly positive or mainly negative?

- Mainly positive experiences

- Mainly negative experiences

- No experience using **care pathway**

7.4 Please describe your most important experiences with the USE of the Liverpool **care pathway**

-……………………………………………….

8.1 If you could change anything about the Liverpool **care pathway**, or how it is applied, what would you change?

[Link with option to download the Liverpool **care pathway**, nursing home version] (<https://shop.iknl.nl/shop/zorgpad-stervensfase-verpleeghuisversie/54902>)

[Example of English version, accessed 31 May 2020, slightly different from Dutch version]

(<http://healthcare.trinityhospice.co.uk/wp-content/uploads/2014/11/D6a-NEoLCP-LCP-1-example-of-the-Liverpool-Care-Pathway-LCP.pdf>)

(examples of care goals in the LCP in supplement 1)

-……………………………………………….

8.2 What would you definitely keep?

-……………………………………………….

8.3 Earlier research shows that the level of knowledge on the part of the care staff is also relevant for the use of the Liverpool **care pathway**. Is the knowledge level of the care staff in your organization sufficient to be able to see positive and negative effects of the use of the pathway for the dying phase?

Choose the option that is BEST REFLECTS the situation in your organization

-Yes; knowledge of palliative care is sufficient, and this supports the effect of the **care pathway** - Yes; knowledge of palliative care is sufficient, but this does not support the effect of the **care pathway**

-No; knowledge of palliative care is insufficient, but this does not affect the effect of the **care pathway**

- No; knowledge of palliative care is insufficient, and this affects the effect of the **care pathway** -No experience using the **care pathway**

9.1 Thank you very much for answering the questions. Would you be willing to answer some additional questions? Do you consent to being approached via e-mail of telephone to make an appointment?

-No

-Yes. Please enter your e-mail address or the telephone number we can use to contact you (you hereby give your consent that we temporarily store this information in the secure environment of the LUMC network) ……………………………….
